# Macroplastic Fate and Transport Modeling: Freshwaters Act as Main Reservoirs

**DOI:** 10.1021/acsestwater.3c00817

**Published:** 2024-05-16

**Authors:** David Mennekes, Yvette A. M. Mellink, Louise J. Schreyers, Tim H. M. van Emmerik, Bernd Nowack

**Affiliations:** † Technology and Society Laboratory, Empa—Swiss Federal Laboratories for Materials Science and Technology, Lerchenfeldstrasse 5, 9014 St. Gallen, Switzerland; ^‡^Hydrology and Environmental Hydraulics Group, ^§^Aquatic Ecology and Water Quality Management Group, Wageningen University, Droevendaalsesteeg 3, 6708 PB Wageningen, The Netherlands

**Keywords:** plastic litter, plastic storage, plastic retention, rivers, lakes, Switzerland

## Abstract

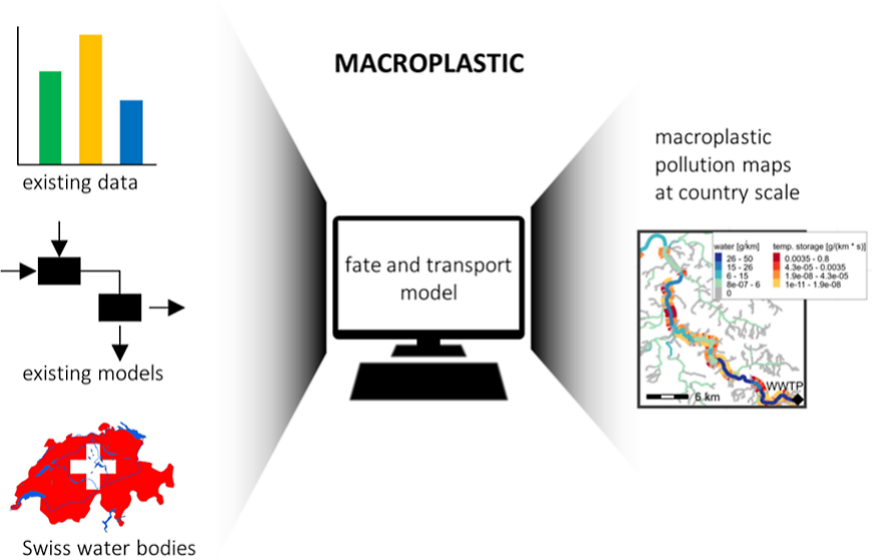

Macroplastic fate and transport in the freshwater environment are of great concern due to the potentially harmful effects of macroplastic on plants, animals, and humans. Here, we present a modeling approach to simulate macroplastic fate and transport at the country scale based on an existing plastic release model. The fate model was parametrized through available monitoring data and results from field experiments and applied to Swiss rivers and lakes. We found that almost all (98%) macroplastic emissions into freshwater remain within Switzerland. After exploring the influences of weirs, retention in rivers, and retention in lakes through a sensitivity analysis, we found a high retention variability across different catchments and within rivers. In all 22 analyzed scenarios for continuous retention along each river bank (i.e., beaching), we found that at least 70% of input emissions into the water bodies would be retained long-term in the catchments (about 200 g per river km and year). Across all catchments, we found a dominance of “continuous retention” through beaching along the entire river length compared with “point retention” at weirs or lakes. Thus, by modeling macroplastic fate and transport on a country level for the first time, we were able to confirm the concept of “rivers as plastic reservoirs” through modeling.

## Abstract 2

A fate model coupled to a high-resolution release model allows us for the first time to predict macroplastic litter concentrations in rivers at the country scale.

## Introduction

The current pace of plastic production is dramatically increasing, which almost inevitably leads to increased plastic emissions into oceans, rivers, and other environmental compartments.^1,2^ Given the harm that plastics can have on ecosystems,^3^ understanding the sources, transport, and sinks of plastics became interesting to many scientists. For macroplastics, particles larger than 5 mm in size, the first modeling studies explored transport processes and sinks in oceans and also aimed to understand sources for ocean pollution.^4−7^ It was found that rivers play a dominant role in plastic transport from land-based sources toward the oceans.^4−6,8^ Regardless of the importance of rivers for plastic pollution, only lately the focus of plastic research shifted toward the sources and, in particular, the role of rivers in transporting and retaining macroplastics.^9^ Existing modeling approaches for oceans simplified the description of freshwater as an input source toward oceans, neglecting the complexity and potential of high macroplastic retention in rivers and lakes.^10−13^

So far, macroplastic transport in rivers has mostly been investigated through point measurements, which provide little information about fate processes or sources of macroplastics.^8,14^ First more in-depth studies revealed highly complex macroplastic distribution, fate, and transport, characterized by a movement of rather small distances with temporal interruptions through beaching or trapping in vegetation.^10,11,13,15,16^ Furthermore, even long-term-accumulated macroplastic might be remobilized during strong flooding events, causing large variations over time in transport rates.^12,17^ Current findings raise the need to close the research gap between release modeling^18^ and long-term sinks in the freshwater environment or transport to the oceans. Currently, macroplastic modeling approaches similar to microplastic models (e.g., nanoDUFLOW^19^ or The Full Multi^20^) are missing. Based on existing knowledge, macroplastic fate and transport are expected to differ from microplastic fate and transport on a country scale.^10,11,21,22^

This article presents a geographically highly resolved macroplastic fate and transport model at the country level. We use the model to analyze how macroplastics emitted into rivers are transported through the river network and finally exported toward neighboring countries based on the example of Switzerland. The potential transport and retention processes were evaluated to estimate both the short-term and long-term retention of macroplastics in rivers and lakes. The model was developed based on a microplastic fate model for rivers and lakes^21^ and parametrized with data from experiments and measurement campaigns for macroplastic. To predict macroplastic concentrations, we used modeled data by a high-resolution macroplastic release model^18^ as input data.

## Methods

**Figure 1:**
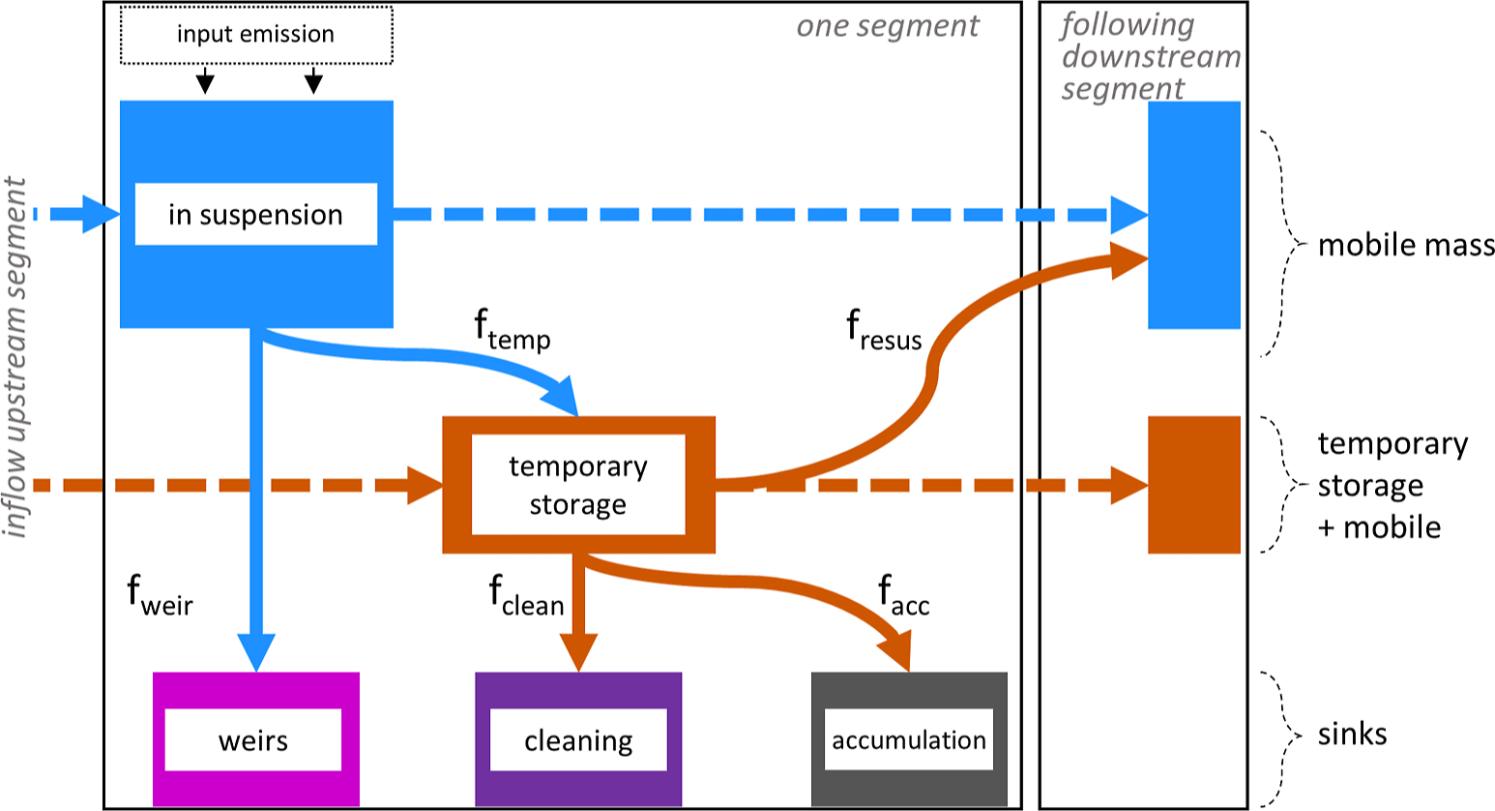
Schematic representation of one model segment, corresponding to one river segment or one lake (including dammed rivers) in the model, with all model parameters and factors describing the allocation of plastics. Mass movement along solid line arrows was calculated based on the corresponding factors. In contrast, transport along dashed line arrows resulted from remaining masses in one compartment after all factor-based mass movements were calculated. The color coding is used for better understanding across all figures.

The presented model predicts macroplastic transport (in mass per time unit) for all waterbodies in Switzerland (an area of about 40 000 km^2^) based on direct macroplastic emissions into freshwaters. This included 35 000 km of river lengths and more than 10 000 lakes, reservoirs, and ponds. We modeled the macroplastic masses individually for each model segment corresponding to a river segment or one lake (including dammed rivers). Within one segment, macroplastics were considered as mobile mass (in suspension), temporarily stored mass, and mass stored in three different final sinks (removed, cleaned, and accumulated; see Figure 1). River segments extended from a few meters to several kilometers in length with an average of 165 m and were typically framed between two river confluences. The final sinks of macroplastics were determined by two main pathways. Macroplastics are directly removed at weirs and dams with hydropower plants (“weirs” in Figure 1), or they are first temporarily stored before being cleaned through anthropogenic activities (“cleaning” in Figure 1) or being long-term accumulated in the river or lake environment (“accumulation” in Figure 1).

Mass movement along the model compartments was described via factors that were calculated individually for each river segment to account for retention, accumulation, cleaning, and removal. In our model, we predicted mass transport and fate under steady-state conditions for the time step of 1 s. All segments were connected according to the water flow direction to predict macroplastic transport and retention across the entire river and lake network of Switzerland. The model was written in R (version 4.1.1) and mainly was built upon an existing microplastic fate and transport model presented by Mennekes and Nowack.^21^ The code is available here: 10.5281/zenodo.10727419 (see the Supporting Information for further details).

The following sections provide a detailed description of the model parameters.

### Retention of Macroplastic in Rivers

Retention through temporary storage was considered one of the most important parts of our modeling, since it influenced sinks and the downstream transport of macroplastics. The probability of temporary storage was calculated for all rivers based on land use (*LU*), discharge (*Q*), and sinuosity (*S*) to cover experimental findings by Newbould et al.^11^ They found that vegetation, rough channel banks, and meander bends in combination with river width were describing the probability of air-filled bottles being trapped in a river. In our model, *LU* in proximity to the rivers was used as a representative value for the channel banks and *Q* was used to represent river width due to better data availability.

For the presented modeling approach, we assumed a simplified flow velocity of 1 m s^–1^ throughout the river network, which can be seen as representative velocity for rivers.^23^ Consequently, macroplastic masses travel in our steady-state modeling 1 m s^–1^. Please note that retention processes based on discharge (*Q*) are indirectly still based on variable flow velocities as presented below.

#### Land Use (*LU*)

Seven land use (*LU*) categories were used as a proxy to describe the potential influence of vegetation on macroplastic transport in the river and the river bank roughness. The *LU* categories were calculated by buffering the river line features (shapefiles by the Swiss Federal Office of Topography swisstopo) up to 200 m depending on the river width (see Supporting Information, Section S2.1.1 for further information). The land use categories were then grouped into four groups of retention potential [none (e.g., water), low (e.g., urban), middle (e.g., rocks), and high (e.g., forest); see Supporting Information, Section S2.1.1] to assign a retention probability based on *LU* (see Supporting Information, Section S2.1.1 for further details).

The probability of temporary storage *p*(*LU*) for a river segment with a length *d* in [m] was calculated by the following equation
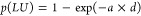
1with *a* being a factor dependent on the retention category (low: 0.00003; middle: 0.00006; and high: 0.00012; see Supporting Information, Section S2.1.1 for calculation). For river segments with mixed land use, we applied a weighted average based on the share of each land use area using eq 1 for each land use category and river segment individually.

#### Discharge (*Q*)

We used average discharge  to represent the large differences in maximum travel distance for plastic bottles observed by experiments in rivers with very different discharge.^10,11,24,25^ Additionally, width, which was found to be an important factor by Newbould et al.,^11^ and discharge are known to positively correlate.^26^ Thus, higher discharge, or larger river width, decreased the probability of macroplastics being long-term stored and increased travel distance.^10,11,24,25^

We assumed that retention probability per segment [*p*(*Q*)] would follow an exponential approximation toward 1 (100% retention) for very small discharges *Q* (in [m^3^ s^–1^])
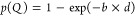
2Here, *d* is the length of a river segment in [m] while *b* is the factor to form the exponential function similar to eq 1. The factor *b* was based on an assumed maximum travel distance (*d*_max_) which was related to average discharges  using results presented in two studies of tracked bottles in rivers by Tramoy et al.^10^ and Newbould et al.^11^ Larger discharge results in smaller values for *b*. A detailed description of the calculation of *b* is given in Supporting Information, Section S2.1.2.

#### Sinuosity (*S*)

Sinuosity (*S*) was correlated negatively with retention probability based on findings by Newbould et al.^11^ For the presented model, we calculate the probability of temporal storage [*p*(*S*)] per 1 m instead of per 10 m by applying a factor of ×0.1 to the provided equation by Newbould et al.^11^ as shown below
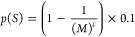
3Here, *M* [-] is the meandering factor calculated by eq 4 as presented by Newbould et al^11^ and *c* is a factor which was set to 0.3 according to Newbould et al.^11^
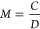
4*C* presents the real river lengths of a river segment in [m] and was observed from the river network file and *D* is the direct distance in [m] between the first and last vertex point of the same river segment. It should be noted that sinuosity values represent the rivers well only when analyzed river segments are long enough. Otherwise, segments are all seen as a straight, nonmeandering river.^11^

#### Combined Temporary Storage Probability

For the combined temporary storage probability in rivers (*f*_temp,river_) of *LU*, *Q*, and *S*, we applied eq 5, which derives the probability of macroplastic mass retention per length unit *p*(*x*_*L*_) (here, 1 m). Equation 5 estimates the average for *LU*, *Q*, and *S*, while a part can be eliminated by setting the probability [*p*(...)] to 0.
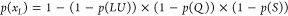
5Finally, the fraction of temporarily stored macroplastics per river segment (*f*_temp,river_) was calculated using eq 6 by accounting for segment length *L* in [m].
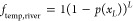
6Here, a negative compound equation accounts for the reduced probability of temporary storage along one river segment with increasing lengths. In other words, losses in the first meters cannot be lost afterward in the following meters. This was taken from the previous model presented by Mennekes and Nowack.^21^

### Retention of Macroplastics in Lakes

**Figure 2:**
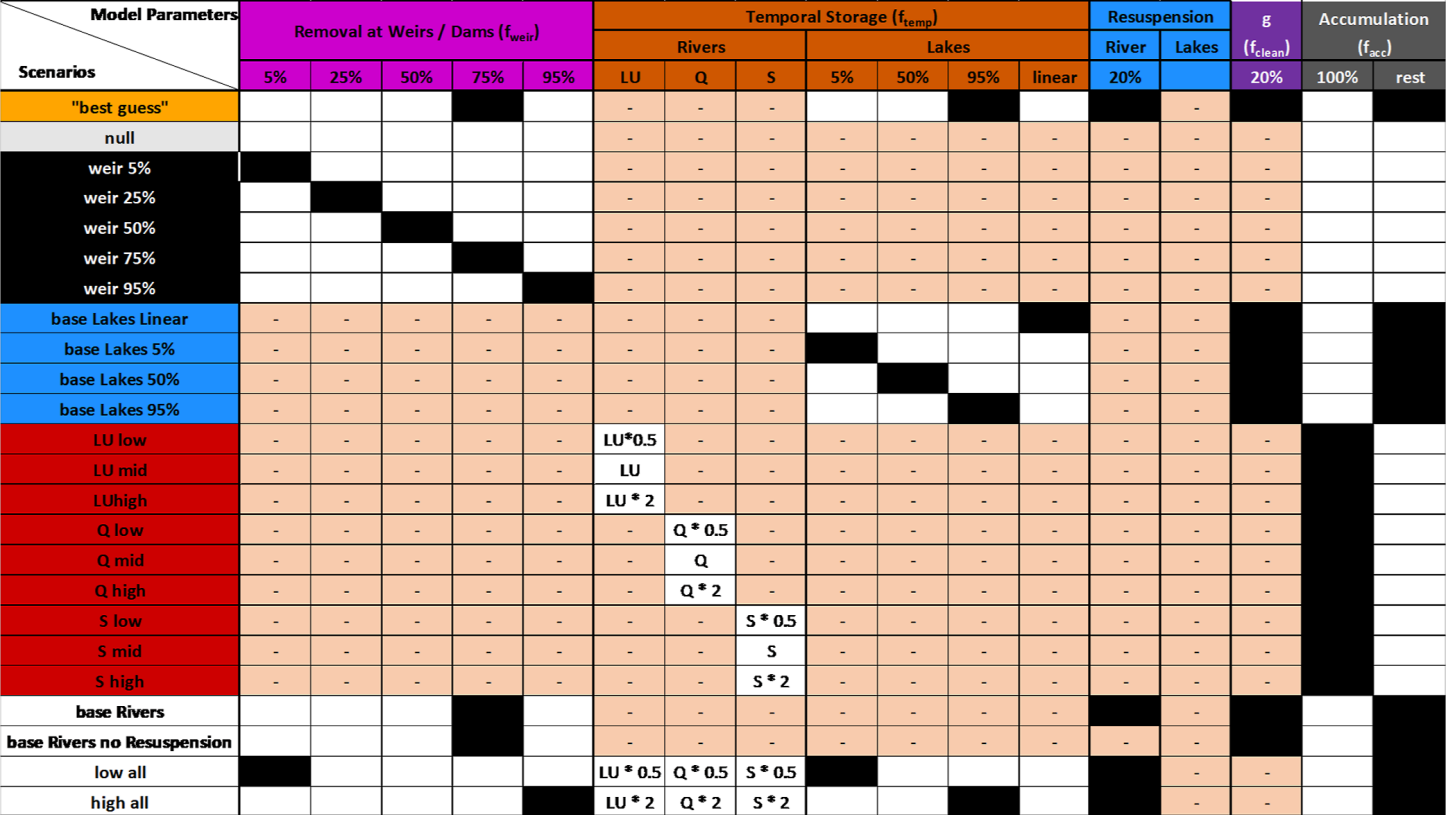
Schematic overview of the different scenarios used and the parameter settings used in the scenarios. Black-filled boxes represent model parameters used for a scenario shown in the rows, while white areas do not apply. Shaded red filled boxes represent model parameters that were eliminated (0) or do not apply to the model scenario. Resuspension for lakes is combined with the temporal storage factor for lakes. The colors of model parameters correspond to the model description in Figure 1, while the colors for the scenarios match with the figures presented in the results.

The temporary storage of macroplastic in lakes (*f*_temp,lake_) was calculated per lake, which included dammed rivers and ponds. However, literature data on macroplastic retention in lakes are largely missing, which is why we used scenarios to explore the potential role of lakes. Scenarios covered different equal temporary storage rates (*f*_temp,lake_) across all lakes (i.e., 5, 50, and 95% of inflowing masses) or a linear function which relates lake-surface area directly to retention (see Figure 2, the following section, and Supporting Information, Section S2.2 for further details).

### Sinks of Macroplastics

In our model, we defined sinks as permanently removed or immobile masses in the river systems. For rivers only, we assumed a direct removal from suspended macroplastic masses at weirs limited to specific weir locations (point sinks) found in the data set “Stauanlagen unter Bundesaufsicht” by the Swiss Federal Office of Energy and swisstopo. Removal rates (*f*_weir_) were a fraction of total bypassing masses and varied in the different scenarios described in Figure 2 (Figure 1). Note that *f*_weir_ was calculated before *f*_temp_.

Other sinks (cleaning and accumulation; Figure 1) were calculated as a fraction of temporarily stored macroplastics per river segment or lake. For rivers, we first accounted for cleaned masses through human activity (*f*_clean_) before calculating other factors affecting the temporary storage (Figure 1). Second, we calculated the mass of resuspended material (*f*_resus_) as a representative value for a yearly average. Finally, the remaining plastic masses in the temporary storage were considered to accumulate long-term in the river environment if not otherwise stated. Here, river cleaning and accumulation can be interpreted as continuous sinks affecting each river segment.

For lakes, resuspension (*f*_resus_) was neglected since the resuspension was considered in the overall fraction of temporary storage (*f*_temp_). Factors were estimated using the sensitivity approach presented in the following section. Similar to rivers, we first predicted cleaned masses before accounting for accumulation.

Finally, if the factor for accumulation (*f*_acc_) in rivers or lakes did not cover the entire remaining mass in the temporal storage, this remaining mass was assumed to be transported downstream to subsequent temporal storage.

### Model Parametrization and Scenarios

We present two general approaches in this article. First, we provide a “best guess” scenario with parameter settings based on derived equations from the literature, as described in the previous sections. Second, we provide a sensitivity analysis of the input parameters to explore how different retention processes influence macroplastic fate by applying the parameter ranges shown in Figure 2. We were able to apply more precise parameters to rivers than lakes in the “best guess” scenarios due to the available literature. Overall, we aimed for a wide range of parameter values, plus we eliminated most parameters while changing only one parameter at a time (ceteris paribus) to isolate the effect of single model parameters. For land use (*LU*), discharge (*Q*), and sinuosity (*S*), we manipulated each value in perspective to the best guess value based on the literature, which we called the “mid” value (see Figure 2). Additionally, we predicted macroplastic masses for scenarios where only one term for the combined temporal storage probability was used (either *LU*, *Q*, or *S* in eq 5), while the other two terms were set to 0. Finally, for “high” scenarios, eq 6 was multiplied by 2, while for “low” scenarios, eq 6 was multiplied by 0.5.

For cleaning (*f*_clean_), we assumed a fixed value of 20% for temporarily stored macroplastics which is in the range of calculations by Tramoy et al.^27^ Furthermore, we presented scenarios with and without cleaning to explore the impact of cleaning. Similarly, we applied a scenario with and without resuspension and a baseline scenario without retention.

### River Network Data and Input Emissions

In the presented model, we used the river network of Switzerland as developed for microplastic modeling in Mennekes and Nowack^21^ and provided by swisstopo. The river network was connected according to the flow direction, including the flows through lakes. Direct macroplastic emissions into the water bodies were extracted from the geographically distributed release model of Kawecki and Nowack.^18^

Based on the input data, this study focuses on the Swiss parts of the catchments. Outflowing macroplastic masses toward the neighboring countries of Switzerland were explicitly modeled for 10 larger rivers, including Rhine and Rhône, while other cross-bordering mass-flows and flows into “dead-end” rivers were monitored separately as flow to “unknown”.

## Results and Discussion

### Transport and Fate on Country Level Based on the “Best Guess” Scenario

**Figure 3:**
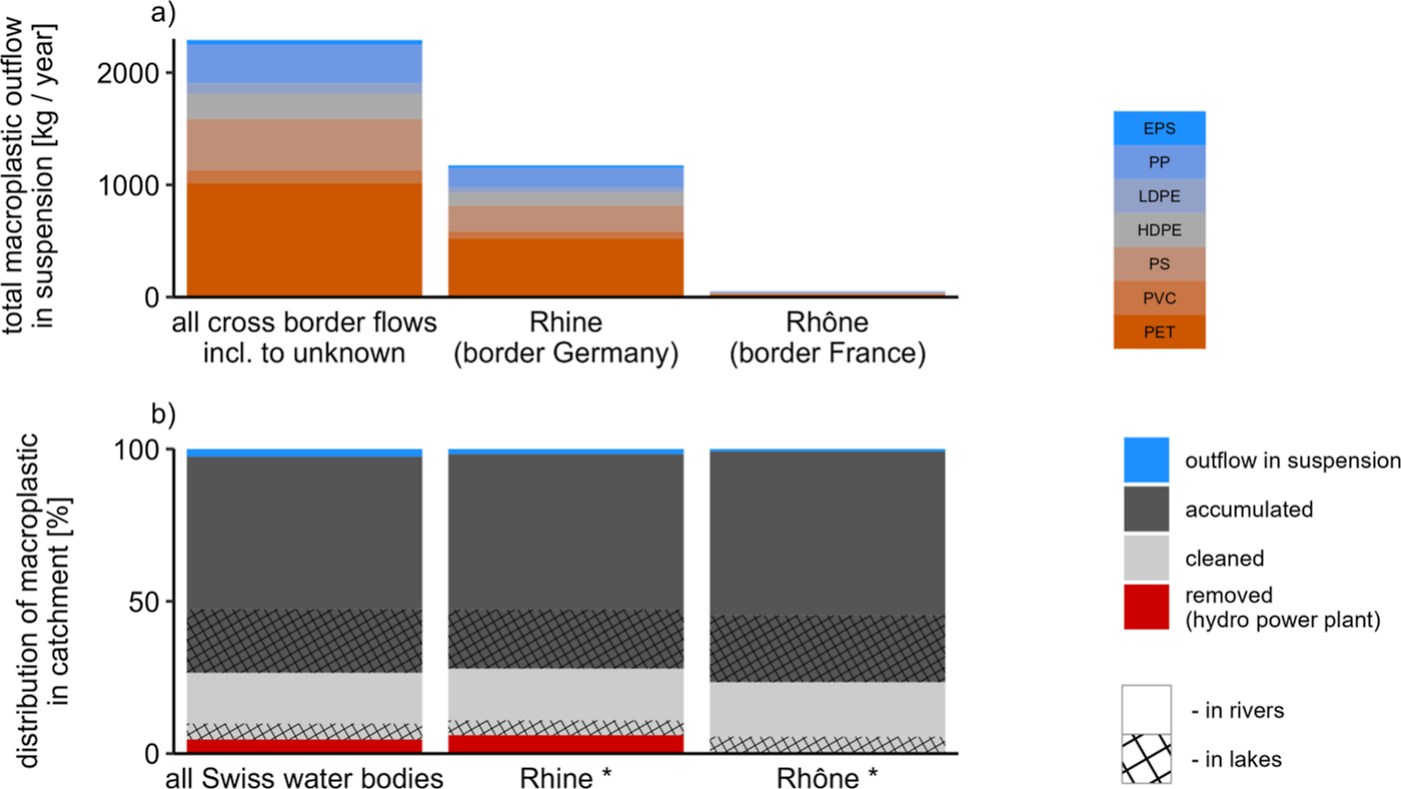
Fate of macroplastic in Switzerland and the rivers Rhine and Rhône based on the “best guess” scenario. (a) Total mass of macroplastic that was predicted to leave Switzerland through one of the major catchments, Rhine or Rhône as well as the overall mass for all Swiss rivers. “Unknown” refers to rivers for which the next downstream segment is “unknown”. Note that the first bar is influenced by 450 kg year^–1^ (about 20%) that flow to “unknown” river segments. (b) Distribution of the macroplastic masses among different sinks and the mass that remains flowing toward the catchment outlet. Here, (a) represents the “outflow in suspension” portion in (b). Additionally, sinks are partly separated into the locations “in rivers” and “in lakes” based on the Swiss river and lake network. * Note: we only considered rivers that discharge toward the catchment outlet.

**Figure 4:**
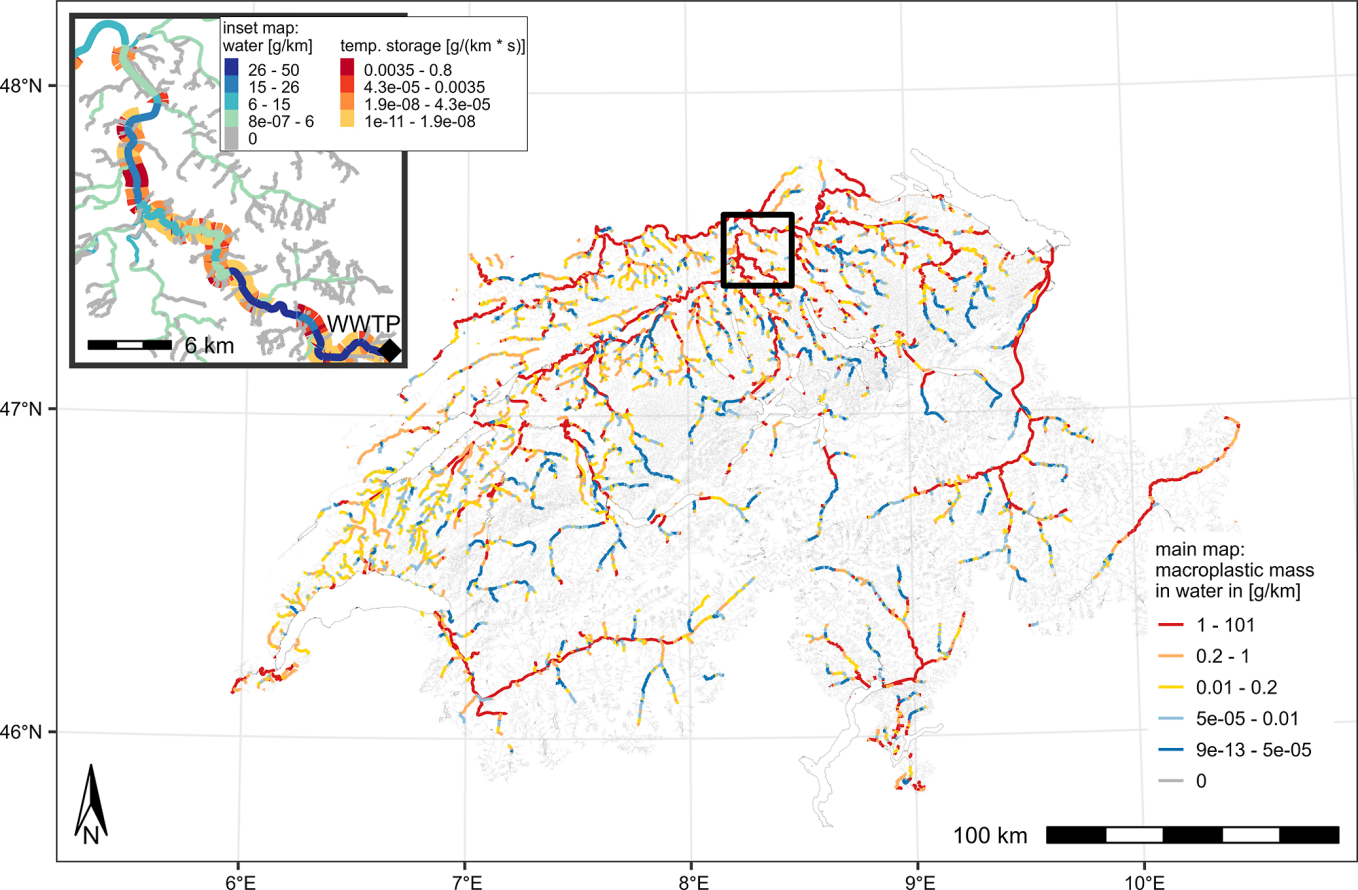
Predicted macroplastic masses in Swiss rivers based on the “best guess” scenario. The inset map shows the masses of macroplastic downstream of the city of Zurich. Additionally, the inset map shows the reduction of macroplastic mass in suspension through removal or temporal storage (e.g., beaching) for the Limmat River (flow direction toward northwest). Note the different legends for the main map and inset map. Furthermore, river bank data is equal for both river sides but might appear different because of overlying colors. The map is based on data from the Swiss Federal Office of Topography, swisstopo.

Across all rivers, we estimated that less than 3% of the about 91 tons year^–1^ macroplastic directly emitted into the rivers^18^ will reach the Swiss border or unknown ends (Figure 3). For the two largest Swiss catchments, Rhine and Rhône, the transport toward the neighboring countries was found to be even lower, with about 1% of a total input of 68 and 6 tons year^–1^, respectively (Swiss parts of the catchments only). If we hypothetically equate the country border with the oceans, these findings are in agreement with Tramoy et al.^27^ and Meijer et al.^5^ who estimated that on a catchment, or global scale, in average, about 2% of plastics enter the oceans. However, we found a high transport variability per catchment (Figures 3 and 4). Small and short rivers were found to transport similar macroplastic masses to neighboring countries than larger catchments due to differences in retention. For instance, the Rhône catchment is 125 times the size of the Breggia catchment but was found to emit only about one-third of the macroplastic masses of the Breggia River catchment toward the neighboring countries. These findings align with results by Meijer et al.^5^ and González-Fernández et al.,^28^ who highlighted that input masses into the oceans are highly driven by sources closely located to the oceans.

Overall, we found that the River Rhine exports the highest macroplastic masses (about 1000 kg year^–1^), while all other rivers analyzed export at least 1 order of magnitude less macroplastics toward the Swiss border (e.g., River Rhône, Figure 3a). The dominant polymer was polyethylene terephthalate (PET) (Figure 3a) due to the dominance of PET in the input emission^18^ and because no differentiation of transport processes among different polymers was applied.

In contrast to our findings for microplastics,^21^ the mass of macroplastic transported via the Rhône River (about 50 kg year^–1^, average discharge  = 338 m^3^ s^–1^) toward France was about in the same order magnitude than other rivers with much lower discharges, e.g., Inn (62 kg year^–1^,  = 20 m^3^ s^–1^) or Breggia (155 kg year^–1^,  = 1.2 m^3^ s^–1^). Additionally, we calculated that about 20% of macroplastic mass was categorized as “flow into unknown”. This included mostly smaller rivers crossing the Swiss border because we analyzed only the 10 largest cross-border rivers individually. Especially, smaller rivers toward France were expected to be contaminated at a close distance to the border,^18^ which reduced the predicted retention and increased the outflowing masses that were captured in the “unknown” compartment. Note that river segments for which the next downstream segment remained unknown, i.e., dead-end rivers, mostly found in karstic regions, were included here.

The spatial distribution of macroplastic masses within Swiss rivers is mainly driven by the littering of consumer products,^29^ which positively correlates with population density due to the model design by Kawecki and Nowack.^18^ For our fate modeling, this becomes clearly visible in a map of modeled macroplastic pollution in rivers, as shown in Figure 4. The western and northern parts of Switzerland, which are characterized by agriculture and more densely populated areas, showed higher values of transported plastic masses in rivers and lakes. In contrast, many parts of the Alps, characterized by less agriculture and low population density, experience very low levels of macroplastic pollution. Overall, we modeled that only 4% of all river segments received plastic pollution. However, it should be stated that transport pathways via wind or from land to water were not considered yet and potentially would increase the number of affected rivers, especially in remote areas. Nevertheless, first transport experiments revealed that mobilization of macroplastics from land toward rivers might be limited unless surfaces had low surface resistance, wind speeds were high, or surface inclination in combination with precipitation favored the direction of transport.^30^

In summary, we estimated that about three-quarters of the input macroplastic emissions were retained through long-term accumulation in river environments. This contrasts with microplastic retention behavior, which was found to be dominated by lakes, e.g., refs (21 and 31–33) but is supported by field observation of macroplastic litter on river banks, e.g., refs (10–12).

### “Rivers as Reservoirs”: Temporary Storage and Long-Term Accumulation

The high retention of macroplastic in rivers observed was mainly caused by temporarily stored and long-term accumulated macroplastic on river banks, e.g., refs (10, 12, 24, and 34). Based on our “best guess” model, we estimated a flux of about 1.8 g s^–1^ from macroplastic in suspension toward temporarily stored macroplastic for the Rhine river catchment, which was about 45 times higher than the estimated outflow in the suspension of the same catchment (0.04 g s^–1^).

As a result of high temporally stored plastic masses, we predicted in the “best guess” scenario high masses of long-term accumulation (without potential resuspension) covering almost the entire input emissions (for Switzerland: 64.8 tons year^–1^; for the Rhine catchment: 48.0 tons year^–1^, Figure 3). However, long-term accumulated plastic can also be interpreted as a potential source of macroplastics that could be remobilized during extreme flood events. Thus, we aimed to represent short-term retention through temporary storage, which included small yearly to biyearly flooding events, while accumulated plastic represented the plastic mass trapped at river banks, sediments, or vegetation over longer time periods.^34^

Larger flood events, on the other hand, can potentially cause high local deposition as well as large-scale remobilization of plastic litter that accumulated over years.^12,15,35,36^ To demonstrate the high reservoir potential of plastics in rivers as stressed by van Emmerik et al.,^34^ we multiplied the accumulation mass in Figure 3 for a 100 year time period, which results in a potentially available median macroplastic mass of 20 kg km^–1^ (value of all polluted river segments). Compared with field data, the nonprofit association hammerdirt^37^ reported for 55 cleanup events on Swiss rivers a median cleaned macroplastic mass of 1 kg km^–1^ river bank. However, cleaning was only performed on one riverside, and most sites were cleaned only one time. Additionally, the cleaned and monitored river banks might often include regularly flooded areas; thus, some cleaned items should be accounted for as temporarily stored plastics since they could have been remobilized in the near future. Overall, the long-term accumulation is difficult to estimate due to the severe effects of strong flooding events. Here, future work of analyzed litter with included date prints could advance our understanding.^15^

### “Point vs Continuous Retention”: Fate on a Single River Scale

**Figure 5:**
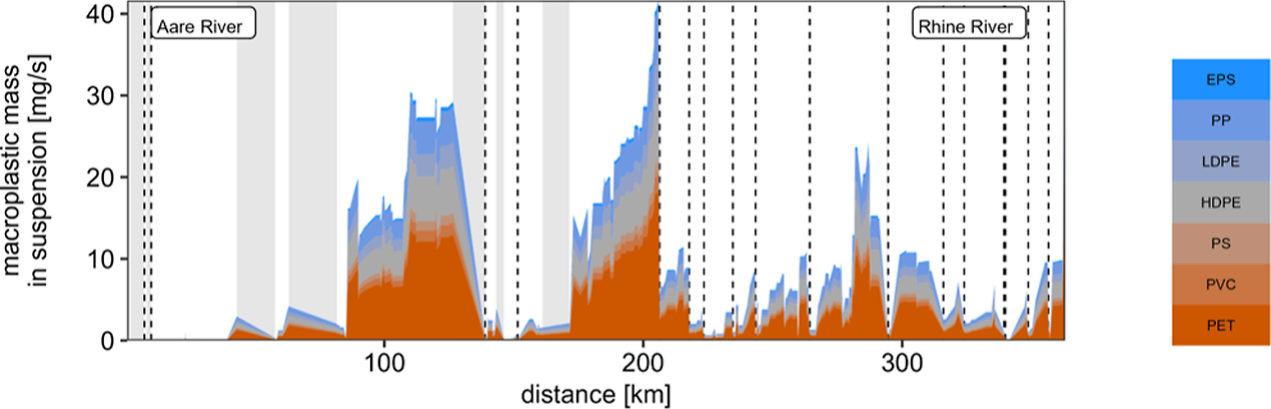
Predicted macroplastic masses along one river from the source of the Aare River until the border with Germany in Basel. Although it is almost invisible, masses increase to about 30 mg s^–1^ very close to the last point on the *x*-axis. The vertical dashed lines mark dams in the Aare and Rhine Rivers. Different colors represent different polymers, while gray-shaded areas show lakes. The distance is based on the Swiss river network file by swisstopo. Please note that the Aare River flows into the Rhine River at kilometer 300.

Focusing our view on one river reveals a high variability of macroplastic masses predicted to be in suspension along the river. This is shown for the profile along the Aare River in Figure 5 and in the inset map in Figure 4, which shows the beaching rates along the Limmat River. In general, we observed two characteristics of retention behavior which we named “point” and “continuous” retention.

Rapid reduction of macroplastic mass in suspension at specific points was mainly caused by weirs and dams and lakes. In the specific example of the Aare River, point retention was observed at the 17 weirs and dams along the river (vertical dashed lines in Figure 5). For each dam in our model, a removal rate of 75% (“best guess” scenario) was assumed, which is in the upper range of values estimated for a part of the Danube catchment by Conversio Market & Strategy GmbH^38^ in collaboration with hydro power plant operators. However, more accurate literature about the removal of macroplastics at dams is widely missing. Instead, when observing the material removed from small hydropower plants, we found macroplastic items as small as candy-wrapping paper being removed by the trash rake (own observation). However, this provides no precise information about bypassing items and masses.

In contrast, continuous retention is a constant flux toward temporary storage along the entire river length, e.g., through beaching, and was described by the parameters *LU*, *Q*, and *S* in our model. When observing the profile of the Aare River in Figure 5, continuous reduction toward temporary storage, followed by potential long-term accumulation or cleaning, seemed to be relatively unimportant compared with weirs; however, the continuous retention processes were found to dominate the overall retention, as also shown in Figure 3. For the main Aare River, as presented in Figure 5 but excluding the Rhine, long-term accumulation in rivers and lakes was estimated to be 1.6 times higher than removal at weirs. For the entire Aare catchment, including all tributaries, removal at weirs was responsible for 6% of the overall retention, while the long-term accumulation in rivers and lakes was found to decrease plastic pollution by 71%. Thus, due to continuous retention, the total transported masses remained relatively constant and did not increase with river distance regardless of the accumulated input emissions. For comparison, the accumulated input emissions, including tributaries, would reach about 2 g s^–1^ for Figure 5 if no retention had been considered.

### Variability and Sensitivity of Macroplastic Retention

**Figure 6:**
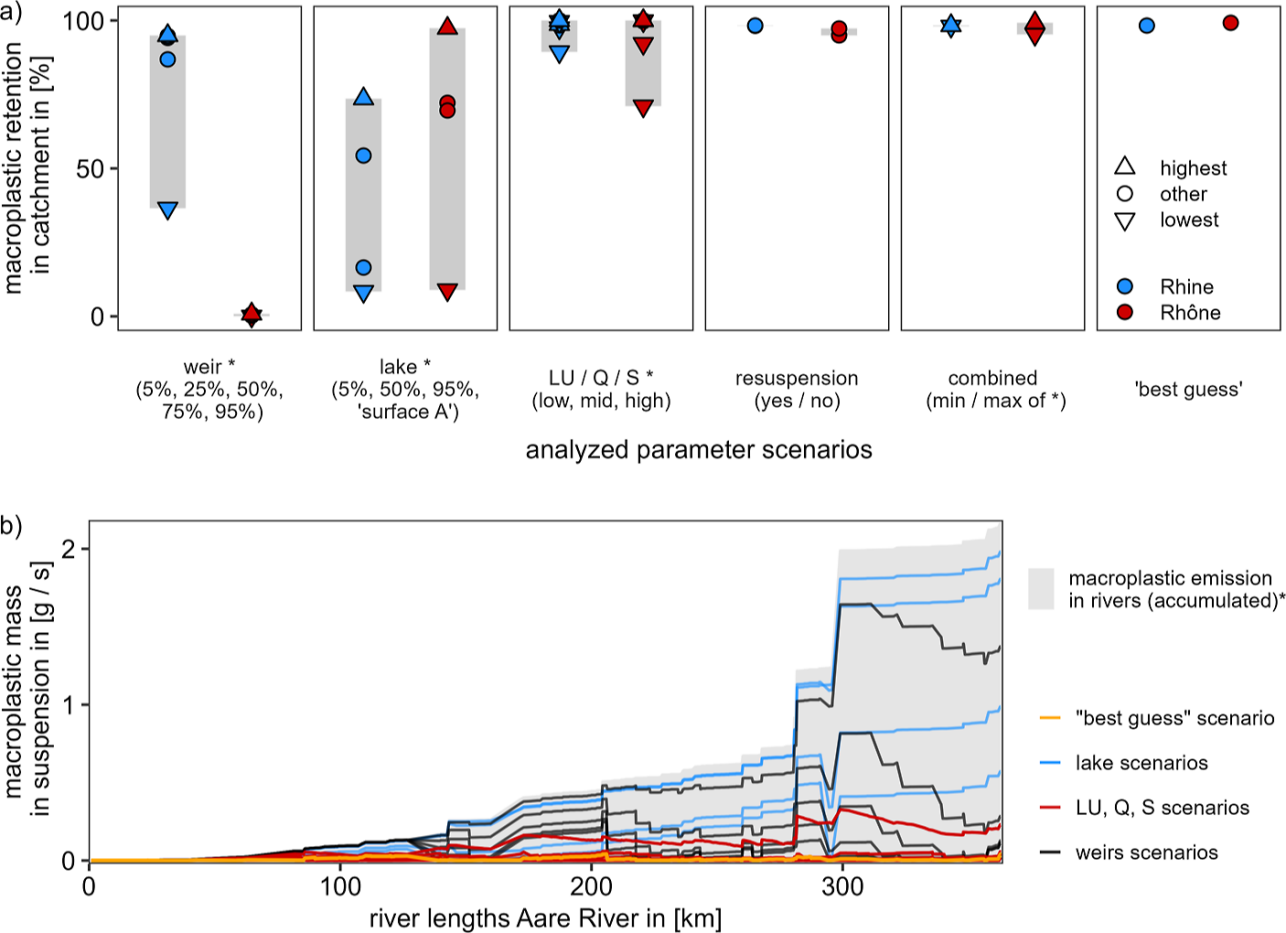
Sensitivity of different simulation scenarios presented in Figure 2 on macroplastic retention in the catchments. (a) Retention of macroplastic in the Rhine and Rhône catchment. The shape of the dots indicates the highest (e.g., “weir 95%“, “*LU* high”) and lowest scenario in regard to the expected retention of each group of scenarios presented in Figure 2. The shape of “other” corresponds to all scenarios which are not covered by “highest” or “lowest”. The gray segments show the observed range of results. High values can be interpreted as low outflowing masses. The combined scenarios include the maximum and minimum retention factors of the scenarios marked by *. The plot of land use (*LU*), discharge (*Q*), and sinuosity (*S*) only represent 3 × 3 points for each catchment since only one parameter was manipulated at a time (see Methods section). A detailed figure is provided in Supporting Information, Section S4. (b) Retention of macroplastic mass in suspension along the River Aare and subcatchment of the Rhine (including River Rhine; confluence at km 300) from source to the border with Germany in Basel which corresponds to the Rhine River in (a). The “best guess” scenario (orange line) is presented in Figure 5 in more detail. * The gray shaded area represents a scenario without any retention, which equals the accumulated input emissions. Differences between the scenarios and the “no retention scenario” for the last *x*-value are shown as percentages in (a) for the entire catchment.

Until today, it currently remains challenging to understand the fate and transport of macroplastic in rivers on a large scale. Here, we explored influencing parameters of macroplastic fate and transport through sensitivity analysis. Figure 6a shows how the different scenarios described in Figure 2 changed the retention in the Rhine and Rhône catchments in Switzerland. Across all scenarios, we observed the most prominent differences between both catchments for the weir scenarios. For the Rhine catchment, a 5% removal scenario at weirs (Figure 2) resulted in an overall retention of about 37%, while other weir scenarios reached retention values up to 95%. In contrast, for the Rhône catchment, the effect of weirs observed through the weir scenarios did not exceed 1% of the overall retention effect (Figure 6a). The low effect of weir scenarios for the Rhône catchment can be explained by the small number of weirs. Thus, mostly small high mountain rivers without plastic input emissions are dammed, and the Rhône as the primary plastic mass transporter is only dammed two times after Lake Geneva, which retains already substantial plastic masses.

Across both catchments, we found land use, discharge, and sinuosity as the parameters with the highest impact on macroplastic retention (high retention in Figure 6a). In contrast, weirs and lakes were differently important for the Rhine and the Rhône catchment (Figure 6a). Finally, we found a comparable low influence of including resuspension compared with excluding it, as shown in Figure 6a. One possible answer could be that resuspension depended on temporary storage, allowing only small masses to be resuspended. Moreover, we speculate that a low sensitivity of resuspension also indicates rather low sensitivities of cleaning toward overall outflowing masses.

Based on the results presented in Figure 6a, we identified three types of sensitivity for the analyzed parameters: (i) we saw “threshold values” above which changes of the parameter only had small effects on the results (e.g., weir scenarios Rhine); (ii) the modeling results were sensitive across the entire parameter range (e.g., lake scenarios); and (iii) the results were less sensitive but the parameter had a high impact [e.g., land use (*LU*), discharge (*Q*), or sinuosity (*S*)]. Throughout all analyzed parameters, we found scenarios of single parameters that were close to the combined scenarios or the “best guess” scenario, which combined all parameters too (Figure 6a). Consequently, all parameters were identified as important for further analysis.

Following (i), for the Rhine catchment, we found that a removal rate of 25% for each weir will show a clear effect of 87% macroplastic retention in the catchment. However, a further increase (i.e., 50–95%) only changed the overall retention in smaller steps from 94.1 to 94.9% (Figure 6a). Here, the remaining macroplastics of about 5% originate from sources close to the catchment outlet (the city Basel). This analysis shows that the presence of weirs as point retention is important, and removal of about 50% could be a good starting value based on our sensitivity analysis and observations by Tramoy et al.^10^ Debris, including plastic litter, must be removed at hydropower plants to protect turbines,^39,40^ although a specific literature about plastic removal is unavailable.

Lakes, as the second important point sink, behaved similar to weirs with equal importance of location. For the Rhône River, Lake Geneva and two reservoirs are located close to the Swiss border (about 20 and 10 km), while for the Rhine, the closest relevant lake to the catchment outlet is further away. Consequently, the lake scenario for Rhône can be interpreted similar to the weir scenario for the Rhine. Here, we interpreted both weirs and sinks in lakes as point sinks with a location-specific impact. However, predicting plastic transport in lakes remains challenging due to its complexity caused by currents and wind and the heterogeneity across the water column.^41−43^ Hence, we started our modeling with different scenarios of retention values but highlighted the need for lake-focused research.

In contrast, for continuous retention parameters (*LU*, *Q*, and *S*) we found high retention values with a low sensitivity [mean = 97% and standard deviation (sd) = 7%; see Figure 6a]. This trend is reinforced when the “low” scenarios for *LU*, *Q*, or *S* that are not considered (mean = 99%, sd = 2%, see Supporting Information, Figure S1). However, it should be considered that the “low” and “high” scenarios for the sensitivity analysis were calculated based on literature values^10,11^ and the range of covered values might be less extreme compared to other scenarios shown in Figure 6a. Our model provides first insights into the complexity and importance of continuous sinks; however, further research is urgently needed to fully understand the macroplastic fate in rivers. Existing experiments and measurements revealed that macroplastic fate processes are highly complex, are driven by stochastic effects, are locally variable, and are not connected to river flow velocity, which is different from findings for microplastics.^10−12,16,44^ Finally, we found that simplifying flow velocity was a justified simplification, as shown by the parameter sensitivity of *Q* in Figure 6a.

Based on our modeling, we conclude that using either *LU*, *Q*, or *S* would be sufficient to describe continuous retention because the parameters partially correlated with each other and showed similar effects in the sensitivity analysis (Figure 6a). For instance, discharge negatively correlates with sinuosity,^26^ and for wider rivers (higher discharge), the impact of vegetation on riverbanks (represented by land use) might become less dominant. Consequently, we suggest simplifying continuous retention with discharge, first because data variability is relatively high and second, the discharge could be helpful to mimic flood events and their higher transport potential.^12,35,45^

Different sensitivities between point sinks and continuous sinks were also visible along the length profile of the River Aare, including the River Rhine, as shown in Figure 6b. Both scenarios focusing on point sinks (lakes and weirs) showed suspended masses, which changed notably with the location of input emissions as well as the location of weirs or lakes (Figure 6b). On the other hand, masses for the scenarios with continuous sinks (depending on the temporal storage, i.e., “best guess”, *LU*, *Q*, and *S*) remain relatively constant over the entire river length (Figure 6b). Hence, maximum transported macroplastic masses in suspension for the “best guess” scenario was 0.04 g s^–1^ [mean: 0.01 g s^–1^ and standard deviation (sd): 0.04 g s^–1^ for contaminated segments], while the maximum value for the *LU*, *Q*, or *S* scenarios was observed for “*LU* low” (Figure 2) with 0.33 g s^–1^. However, across all scenarios of *LU*, *Q*, or *S*, the average maximum values were lower (0.06 g s^–1^) with maximum values as low as 0.002 g s^–1^. More or less constant macroplastic masses were also found by measurements along the entire Rhine until the river mouth, supporting the concept of continuous retention.^46^ Overall, it would be beneficial to better understand the complex processes of temporary and long-term storage in river systems. Nevertheless, extreme flooding events might interrupt a system of continuous sinks and stable concentration along a river, which causes potentially high variability on (temporal) transported and stored plastic.^12,35^

### Comparison with Field Data

We compared our model results to two types of measurement data: first, measurements of floating macroplastic at the water surface as conducted by Kuizenga et al.^46^ for the Rhine in Switzerland and second, measurements of beached litter at river banks and lakesides as observed by “hammerdirt!” (www.hammerdirt.ch).

When comparing measurements in the Rhine for the locations Koblenz (CH) and Basel (CH) with our modeling data, the model predicted masses are about 1 order of magnitude lower than water surface observations by Kuizenga et al.^46^ (about 1 tons year^–1^ compared with 12 tons year^–1^, Figure 3; standard deviation weight per item: 5.5 g; please, refer to Supporting Information, Section S3 for transforming items counted into mass). Comparing the monitored masses by Kuizenga et al.^46^ with currently estimated input emissions into the Rhine catchment (91 tons year^–1^^18^), we found a retention that would be clearly lower (87%) than previously observed (98%, our work, Tramoy et al.,^10^ and Meijer et al.^5^). Moreover, the proportional retention can be even lower when considering that measurements by Kuizenga et al.^46^ might have underestimated item numbers because the measurements were performed under low flow conditions, no peak season for recreational activity (March) and at the water surface only.^12,17,47−50^ Additionally, macroplastic is expected to be transported with a time delay from the sources toward the catchment outlet due to temporary storage/reservoir.^34,49^ Hence, today we observe plastics in the river which were released in the past, when plastic emissions were presumably lower compared with today.^2^

A potential explanation for the difference between modeled and observed plastic masses could be a different distribution of the emissions than currently modeled (favoring the area next to the catchment outlet) or a much higher catchment-wide input emission of about 600 tons year^–1^ to match expected retention percentages in the catchment. This would be 1 order of magnitude higher than direct emissions into the water estimated by Kawecki and Nowack.^18^ Here, direct emission means that the release model by Kawecki and Nowack^18^ does not include an estimate of macroplastics indirectly released into surface waters via transport from soils. Therefore, a coupled soil–water macroplastic fate model would be needed. However, the current understanding is that transport over land (e.g., soils or grassland) is less likely.^51^ On the other hand, large overland transport rates might be possible through drainage systems and sealed surfaces with low surface resistance.

Other studies estimate the yearly transported mass for the Rhine at the river mouth in The Netherlands from 0.5 to 60 tons year^–1^ based on different discharge scenarios.^5,46,52,53^ Assuming a stable mass conservation along river length,^46^ masses differ by about 1 order of magnitude compared to our modeled masses in Basel (about 1 tons year^–1^, Figure 3). Additionally, it should be considered that observations by Kuizenga et al.^46^ might underestimate mass transport since the floating surface litter is only one part of the total mass transport.^47−49^

Whereas polymer-specific parameters are currently not included in the model, most monitoring studies in rivers found a dominance of *PO soft* (soft polyolefins) which represents foils, plastic bags, or soft pieces of plastic (mainly, LDPE and PP).^28,46,47,49,52^ In contrast to our polymer-unspecific modeling, we expect that only about one-fourth of the plastics are categorized as *PO soft*. A possible explanation could be an overestimation of PET input emissions in comparison with other polymer input masses as predicted in the model by Kawecki and Nowack,^29^ which forms the basis of our fate model. Less likely is a substantial underestimation by monitoring studies of PET-macroplastic related to an invisible transport at the bottom of the water column.^47^ Finally, experiments presented in a baseline study by Ivar do Sul et al.^54^ and observational data by Hauk et al.^35^ suggest different retention behaviors of different items (or polymers). Hence, first results suggest that plastic bags (*PO soft*) are more likely retained in rivers within a short distance than for instance plastic bottles (PET).^54^

For comparing our modeling data with collection data of beached macroplastic reported by hammerdirt,^37^ we selected three observed river banks with more than one measurement round from the data set (two at the Aare River and one at the Limmat River). To calculate the accumulation per time unit, we assumed that all litter collected on the later collection campaign was beached after the first cleanup. Masses of observed beached or accumulated litter were at least one order of magnitude higher compared with modeling data during the same period. Thus, hammerdirt^37^ observed 0.1, 1.3 g m^–1^ (both Aare River), and 0.4 g m^–1^ (Limmat River) of beached macroplastics (per river lengths) after the period of 32, 197, and 290 days, respectively. While using longer observation periods would result in higher accumulated masses in our model due to the modeling approach, these findings cannot be confirmed by the selected observational data.^37^ Additionally, collected items at all three sites suggested a low share of PET since almost no drinking bottles were found (1 out of 123 items^37^).

As shown above, there are differences between the monitoring data and modeling results. The model is intended to predict yearly averages based on a nationwide release model, which was regionalized using several proxies. Instead, monitoring data captured a snapshot of the real world, which is likely to display high local variability. Additionally, measuring macroplastics is challenging regardless of the size due to issues such as underestimating nonfloating plastics and the high variability of beached items across multiple locations.^16,47−49^ Finally, river cleanup campaigns usually target only selected sections of the river banks or lake shores, in particular those easily accessible and known to be highly polluted by plastic. However, this is beyond the possible spatial resolution of our fate model, which provides average masses over longer distances.

## Concluding Remarks

To summarize, the presented model showed a high potential for further exploring macroplastic fate and transport in an aquatic environment. Especially, future experiments following the recent examples might bring better perspectives in order to advance in interaction with models our process understanding.^10,11,13,24,25^ Based on our results, we suggest the following improvements to macroplastic fate modeling.

(i) The model retention processes are not yet able to distinguish among likely differences in behavior for different items (e.g., films vs bottles)^35^ due to missing process understanding and experimental data. This would be needed to provide better results for different groups of macroplastic. The underlying release model by Kawecki and Nowack^29^ does contain more detailed data on the type of plastic released, which could also be useful for the fate model. Additionally, the process of temporary storage, including the time delay, should be further improved.
(ii) Temporal resolution is currently missing in our model but would be desirable in further model implementation due to observed differences between extreme floods and average flow conditions.^35^ Here, we suggest dynamic input emissions, which would extend our current steady-state modeling.
(iii) The macroplastic release processes into the environment should be further investigated as they form the basis for quantifying absolute concentrations in our model. Kawecki and Nowack^18^ provided the first high-resolution emission model with type of items, polymer type and geographic distribution. However, the quantification of emissions into the environment remains highly uncertain which might have caused an overestimation of the mass flows of PET compared to beaching or floating litter observations.^37,46^

We conclude that the fate and transport of macroplastics might substantially differ from microplastic behavior. Especially, the high retention of macroplastics in rivers, previously introduced as “Rivers as Plastic Reservoirs”,^34^ was a dominant process on a country scale. In alignment with this, we found that “continuous retention” through beaching along all river segments dominates the retention compared with “point retention” at specific locations such as removal at weirs. This remained also valid under different scenarios tested in a parameter sensitivity analysis. Comparing our modeling results directly with monitoring studies remains challenging due to various reasons (for instance: high variability of monitoring data coverage of different processes). However, the overall retention on a country scale (98%) is in alignment with other large-scale macroplastic modeling studies.^5,27^ Finally, we urgently call for action to better understand the origin, fate, and transport of plastic pollution in rivers to be able to decrease plastic pollution and distribution in the environment.

## Data Availability

The code is available here: 10.5281/zenodo.10727419.

## Supporting Information Available

Additional method details, code description, and results (PDF)
