# Supplementary material for: Macroplastic Fate and Transport Modeling: Freshwaters Act as Main Reservoirs
Source: ACS ES T Water. 2024 May 16;4(6):2470–81. doi: 10.1021/acsestwater.3c00817 (PMC11186010; doi:10.1021/acsestwater.3c00817)
Supplement: Supplementary file 1 — ew3c00817_si_001.pdf [file ew3c00817_si_001.pdf]

Supporting Information

Macroplastic fate and transport modeling: freshwaters act as main reservoirs

David Mennekes,† Yvette A. M. Mellink,‡ Louise J. Schreyers,‡ Tim H.M. van Emmerik,‡ and Bernd Nowack*,†

†*Empa - Swiss Federal Laboratories for Materials Science and Technology, Technology and Society Laboratory, Lerchenfeldstrasse 5, 9014 St. Gallen, Switzerland*

‡*Wageningen University, Hydrology and Environmental Hydraulics Group, Droevendaalsesteeg 3, 6708 PB Wageningen, The Netherlands*

E-mail: bernd.nowack@empa.ch

The supplemental information provides further information on how to calculate model parameters as well as further figures for the Results and Discussion section.

1 Description code

Below, we explain the purpose of each R script.

An example river network with code is presented in Mennekes and Nowack[1].

Please note that scripts for the main model are numbered 03.0. Any script listed beforehand is needed to prepare the data for running the model. Scripts listed after 03.0 are used for presentation purposes. The numbering is aligned with the previous model presented for microplastics[1].

* to *** show the importance of the script from low to high.

- `01_collect_river_data.R`

  This script is not needed in all cases. Here, contamination data is transferred to one file only and the connection ("flow to") is added. In case your data does provide this data, please skip this script.

- `02.1_outflows_country.R`

  We created river line features that are connected to the last line feature, which is still in Switzerland, in terms of down-flowing rivers. The idea is to have a line feature without any interactions of sedimentation etc., to obtain the outflow of microplastics from the country. Additionally, we added one outflow container for all rivers that are missing a next downstream connection either because they are small border crossing rivers or because they are "dead-end" rivers.

- `02.2_add_further_information.R`

  Add elevation information to the river network. This step is needed for estimating the flow velocity. Please note not all data is available for this step due to data size / copyright by other parties. Please get in touch with the authors for further information.

- `02.3_get_flow_velocities.R`

  This script will add an average water flow velocity to each river segment. For the

Swiss river network, we connected the data with an older data set, which included modeled average flow velocities. Furthermore, we added measured values when possible. Please note not all data is available for this step due to data size / copyright by other parties. Please contact the authors for further information.

- `02.4_get_max_flow_length.R`

  This script determines how many time steps are needed to establish an equilibrium in the system. We recommend a few time steps more than the longest river is long in time steps. In our case, we use 800 time steps.

- `02.5_contamination_ms.R` *

  In this script, we change contamination values from yearly values to values per second regarding the flow velocity. In other words, the values describe how much plastic is passed on per second.

- `02.5b_contamination_ms.R` *

  This script is needed to gather further information for the macroplastic factors. E.g. landuse, meandering etc. Original input data for Switzerland is available via the swisstopo webpage.

- `02.6_factors_lakes_MaP.R` *

  With this script, we determined the surface area depended on lake sedimentation rates. You will find the equations and curves for each polymer. However, this script is not connected to any other script but was used only to derive the factors.

- `02.6_factors_rivers_MaP.R` **

  Here, the factors for rivers are defined based on the 5 % approach described in the paper.

- `02.7_factors.R` ***

Here, the factors for rivers and lakes are applied to the river network based on lake surface area or river lengths. The script determines the scenarios which will be calculated. If factors are not determined differently, this script is necessary!

- `03.0_run_materialflow_river.R` ***

  This scripts run the actual model.

- `03.1_printMaps.R` **

  This script prepares the output files of `03.1_printMaps.R` for further use. One can select columns (information) of interest, which will be exported as a .gpkg or Rdata file.

- `04_...`

  These scripts are used to present the data (numbers or plots).

2 Additional information for Methods

2.1 Fraction of temporal storage (f_{temp}) in Rivers

Please note that some parts are also presented in the article itself. This was done for better readability and understanding. Similar to Mennekes and Nowack[1] f_{temp} was calculated by the negative compound interest equation (eq. (1)), which transfers a reduction per length (m^{-1}) into a reduction valid for an entire segment of multiple meters.

$$f_{\text{temp, river}} = 1(1 - p(x_L))^L \tag{1}$$

where L is the lengths of a segment in m an $p(x_L)$ is the probability of sedimentation per m calculated by the retention probability based on land use (LU), discharge (Q) and sinuosity (S) as shown in eq. (2):

$$p(x_L) = 1 - (1 - p(LU)) * (1 - p(Q)) * (1 - p(S)) \tag{2}$$

Equation (2) is based on[2] who used slightly different input data. Below, each part of eq. (2) will be further explained.

2.1.1 Land Use (LU)

Land use for Switzerland was grouped into the following categories using available data sets by the Swiss Federal Office of Topography swisstopo (swissTLMRegio_LandCover.shp): rocks, agriculture, glacier, water, urban, forest and unknown (this is mostly agriculture land as we could observe from satellite data)
In a second step, we buffered all rivers with 10 m, 100 m and 200 m and derived the proportional distribution of each land use categories for each buffer segment which correspond to the river segment. We used the 10 m buffer as long as a maximum of half of the land coverage was covered by water. For other river segments, we applied the 100 m

or 200 m buffer following the same process.

In a second step, we derived LU dependent retention by assuming a maximum travel distance of macroplastics for the four groups of retention potential (none, low, middle, and high). The assumed maximum travel distances were chosen in perspective with the results presented in the experiments by Tramoy et al.[3] and Newbould et al.[2] and to "unlimited", 100 km, 50 km, and 25 km for none, low, middle, and high retention respectively. Finally, we used an exponential function presented in eq. (3) for maximum travel distances in order to account for high chances of retention close to the input (starting) point and lower retention probability further away.

$$p(LU) = 1 - exp(-a * d) \tag{3}$$

Here, $p(LU)$ is the retention per river segment and d is the river segment length in [m]. To derive factor a in eq. 1 in the article (eq. (3) in the SI) we used the assumed maximum distances per land use category (Table 1). Please note that the equation does not apply to the category "none" since we assumed no retention. The other three categories can be translated into a maximum travel distance assuming that at the maximum travel distance, the remaining mass is below 5 % of initial mass. Following this assumption, we resolved eq. (3) by setting $p(LU) = 0.95$. Here, we use 0.95 because we multiply the factor ($p(LU)$) with the mass in solution to derive the temporarily stored mass (see the figure in the article). Afterward, the masses will be subtracted; in other words, the remaining mass is 5 % of the original mass.

With $p(LU) = 0.95$ and d set to the maximum distance in [m] corresponding to the retention categories presented in Table 1 we derived the values for a. This is needed, in order to use eq. (3) for each river segment with individual section length d.

LU categories were assigned to the different retention categories according to an expert opinion survey among the group of authors. The following associations of land use with retention factor were used in the model (see Table 1).

Table 1: Categories of land use (LU) used to predict the retention of moving macroplastics in suspension towards temporally stored macroplastics. The assignment of land use categories to retention levels was done by the author's expert experiences. The 5 % distance describes the theoretical distance after which only 5 % of the initial mass is remaining following eq. 1 in the article (flow velocity = $1\,m\,s^{-1}$). Please note that f_{temp} is beside LU, based on discharge and sinuosity.
* see article for the equation

retention level	5 % distance	factor *a* for eq. 1*	land use category
none			water, glacier
low	100 km	0.00003	urban
middle	50 km	0.00006	rocks, unknown (mostly agriculture)
high	25 km	0.00012	forest, agriculture

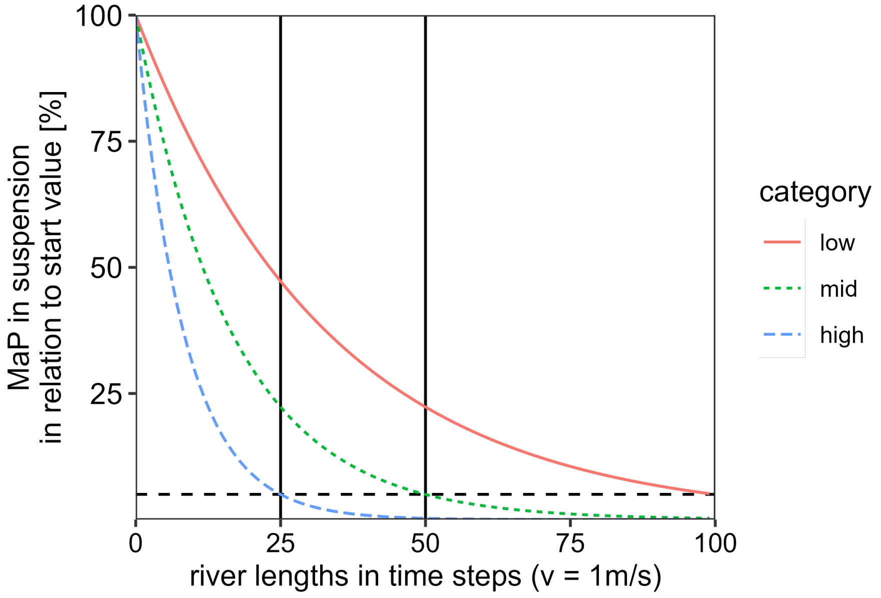

Figure 1: Retention of macroplastic according to the three retention categories "low", "middle" and "high" for land use factors.

2.1.2 Discharge (Q)

The equation for maximum travel distance (eq. (4)) was based on observation data by Tramoy et al.[3] and Newbould et al.[2]. Based on existing data, the maximum travel distance $d_{max}(Q)$ is defined as the distance where 5 % of the initial mass is left in dependency of the discharge Q in $[m^3\,s^{-1}]$. In other words, 95 % of the mass is retained in the temporary storage (see explanation in the article). The maximum distance was derived

using the following equation:

$$d_{max}(Q_{\overline{x}}) = \begin{cases} 3333 * Q_{\overline{x}} & \text{if } Q_{\overline{x}} \leq 0.3 \\ 330 * Q_{\overline{x}} + 901 & \text{if } Q_{\overline{x}} > 0.3 \end{cases} \quad (4)$$

Here, the first case was derived from[2] who found for a small river with a discharge of 0.3 $m^3 s^{-1}$ (width: 4 m) a maximum travel distance of 1 km. However, most bottles traveled much shorter with an average travel distance of about 230 m. This means the maximum travel distance is a rather conservative approach. The second case in eq. (4) was based on Tramoy et al.[3] who found that their tracked bottles would travel less than 100 km during a ten-year flooding event with a discharge about fivefold higher than normal discharge. As a conservative travel range, we applied the maximum travel distance observed during the flooding event for non-flooding discharge. Consequently, the probability of temporal storage might be underestimated, however, Tramoy et al.[3] only observes a limited time window which might not cover the final and last travel point of the observed bottles.

Based on the assumption that at d_{max} 95 % of the input mass is retained we can use the exponential function for the retention factor $p(Q)$ stated in the article and repeated here:

$$p(Q) = 1 - \exp(-b * d) \quad (5)$$

While d is the river segment length in [m] b is a factor based on d_{max}. Therefore, the given values can be entered in eq. (5) as followed:

$$0.95 = 1 - \exp(-b * d_{max}(Q_{\overline{x}})) \quad (6)$$

Now, eq. (6) can be rearranged to eq. (7) to calculate b.

$$b = -\frac{\ln 0.05}{d_{max}(Q_{\overline{x}})} \quad (7)$$

Finally, b was calculated for each single river segment and then applied to eq. (5) to calcu-

late discharge-based temporary storage [m^{-1}] ($p(Q)$) individually for each river segment.

2.2 Temporal storage (f_{temp}) for lakes

The most simple approach is a single fraction per lake. Here, we used scenarios with 5 %, 50 % and 95 % of all inflowing and emitted plastics into a lake being temporarily stored. Additionally, we used a lake size-dependent scenario using the lake surface area in [m^2] as an approximation for the lake size. Based on a linear equations we set two fixed f_{temp} of 0 % and 95 % for the surface areas of A = 0 km^2 and A = 600 km^2 (surface area of the largest lake in Switzerland: 580 km^2). The corresponding linear equation is:

$$f_{temp,\, lakes\, linear} = A * 1.58 \times 10^{-9} \tag{8}$$

with A in m^2 and $f_{temp,\, lakes\, linear}$ being a factor between 0 and 1.

3 Transforming observed macroplastic item numbers into mass

For comparison we transferred reported item numbers (total plastic items: Basel = 142 items/hour (PO soft = 129 items/hour), Koblenz (CH): 129 items/hour (PO soft = 36 items/hour),[4]) into masses using a combined data set with data by Vriend et al.[5] and Rijkswaterstaat[6] (mean all categories: 9 g/item (sd: 42 g), mean PO soft: 2 g/item (sd: 6 g). Further information is available upon request.

4 Additional Figures for Results and Discussion

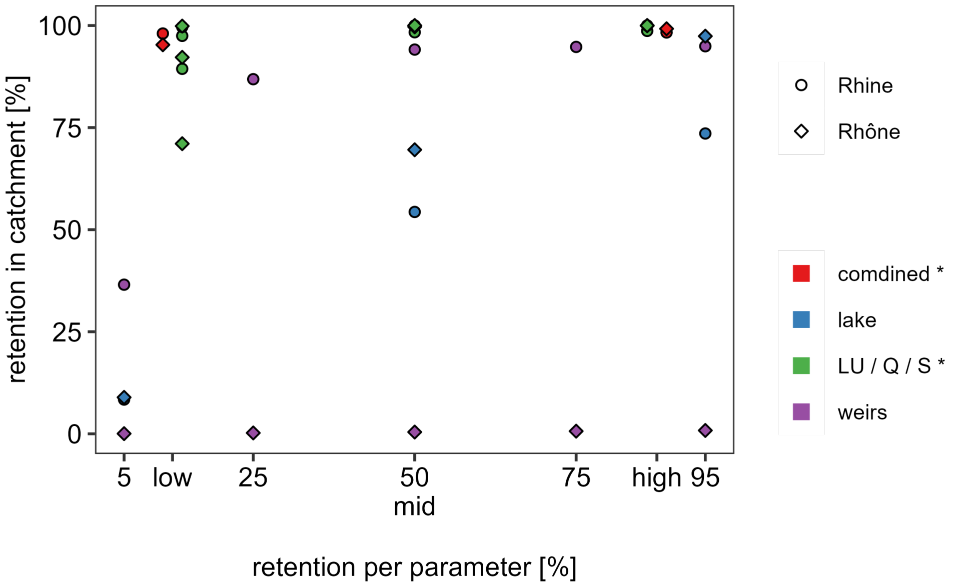

Figure 2: Retention of macroplastic emission in the catchment (compared with total input emissions) in dependency of the scenarios. The x-axis shows the retention happening for the parameters, for instance, 5 % for weirs or lakes represents a scenario in which at each weir / lake 5 % of the mass are removed. * Note: the "combined" and "LU / Q / S" scenarios a located on the x-axis to the discrete values "low", "mid", "high" which were added to the continuous axis for better comparison.

References

(1) Mennekes, D.; Nowack, B. Predicting microplastic masses in river networks with high spatial resolution at country level. *Nature Water* **2023**, *1*, 523–533.

(2) Newbould, R. A.; Powell, D. M.; Whelan, M. J. Macroplastic Debris Transfer in Rivers: A Travel Distance Approach. *Frontiers in Water* **2021**, *3*, 1–14.

(3) Tramoy, R.; Gasperi, J.; Colasse, L.; Silvestre, M.; Dubois, P.; Noûs, C.; Tassin, B. Transfer dynamics of macroplastics in estuaries – New insights from the Seine estuary: Part 2. Short-term dynamics based on GPS-trackers. *Marine Pollution Bulletin* **2020**, *160*, 111566.

(4) Kuizenga, B.; Tasseron, P. F.; Wendt-Potthoff, K.; van Emmerik, T. H. M. From source to sea: Floating macroplastic transport along the Rhine river. *Frontiers in Environmental Science* **2023**, *11*.

(5) Vriend, P.; van Calcar, C.; Kooi, M.; Landman, H.; Pikaar, R.; van Emmerik, T. Rapid Assessment of Floating Macroplastic Transport in the Rhine. *Frontiers in Marine Science* **2020**, *7*, 10.

(6) Rijkswaterstaat . Kentallen dataset rivieren. 2023; `https://www.afvalcirculair.nl/zwerfafval-microplastics/kennisbibliotheek/rivier/kentallen-dataset-drijvend-zwerfafval/`.
